# A reference method for determining the total allergenic protein content in a processed food: the case of milk in cookies as proof of concept

**DOI:** 10.1007/s00216-020-02959-0

**Published:** 2020-10-02

**Authors:** Maria José Martinez-Esteso, Gavin O’Connor, Jørgen Nørgaard, Andreas Breidbach, Marcel Brohée, Elena Cubero-Leon, Chiara Nitride, Piotr Robouch, Hendrik Emons

**Affiliations:** 1 European Commission, Joint Research Centre, Retieseweg 111, 2440 Geel, Belgium; 2 Present Address: Departamento de Agrochímica y Biochímica, University of Alicante, Carretera de San Vicente del Raspeig s/n, 03690 San Vicente del Raspeig, Alicante Spain; 3 Present Address: Physikalisch-Technische Bundesanstalt, Bundesallee 100, 38116 Brunswick, Germany; 4 Present Address: Department of Agriculture, University of Naples ‘Federico II’, 80138 Naples, Italy

**Keywords:** Food allergen detection, Metrological traceability, Mass spectrometry, Peptide standards, Protein quantification, Processed food

## Abstract

The establishment of a reference method for the determination of the allergen protein content in a processed food material has been explored. An analytical approach was developed to enable the comparability of food allergen measurement results expressed in a decision-relevant manner. A proof of concept is here presented, resulting in quantity values for the common measurand, namely ‘mass of total allergen protein per mass of food’. The quantities are determined with SI traceability to enable the comparability of reported results. A method for the quantification of total milk protein content in an incurred baked food at a concentration level clinically relevant is presented. The strategy on how to obtain the final analytical result is outlined. Challenges associated with this method are discussed, in particular the optimal extraction of the marker proteins, the complete digestion and release of the peptides in an equimolar fashion, the use of conversion factors to translate the amount of measured proteins into total milk protein and the estimation of the uncertainty contributions as well as of the combined uncertainty of the final result. The implementation of such a reference method for the determination of the total allergen content in a processed food is an important step, which will provide comparable measurement data of relevance to risk assessors.

**Electronic supplementary material:**

The online version of this article (10.1007/s00216-020-02959-0) contains supplementary material, which is available to authorized users.

**Figure:**
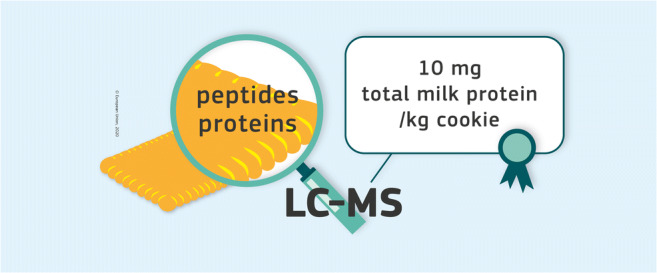
Graphical abstract

## Introduction

European Union (EU) Regulation No. 1169/2011 requires the correct labelling of 14 priority allergenic ingredients in foodstuff to guide allergen sufferers in making informed choices [1]. Any hazard arising from the unintentional presence in food of those substances, for instance as a result of cross-contamination, must conform to the provisions of Regulation (EC) No. 178/2002 (General Food Law) [2]. Precautionary allergen labelling (PAL), the use of ‘may contain’ statements, in such cases is voluntary although Article 36 of Regulation (EU) No. 1169/2011 entitles the European Commission to adopt law on information on the unintentional presence in food of substances causing allergic or intolerance-based reactions. However, the voluntary labelling of the likely unintentional presence of those substances, for instance as a result of cross-contamination, is permitted by the use of a ‘may contain’ statement as a precautionary allergen labelling (PAL). PAL is based on provisions of the Food Information to Consumers contained in Regulation (EU) No. 1169/2011 and on Regulation (EC) No. 178/2002 (General Food Law). These require food business organizations (FBOs) to implement appropriate risk assessment and risk management procedures to ensure that food placed on the market within the EU is safe. Article 50 of the General Food Law established a Rapid Alert System for Food and Feed (RASFF). It obliges EU Member States to inform the European Commission about the existence of a serious risk or indirect risk to human health deriving from food or feed. A number of such alerts still occur due to the omission or incorrect labelling of the presence of one or more of the 14 allergens on foodstuff labels. However, the proliferation of PAL and its inconsistent application is confusing to consumers. The ‘may contain’ labelling should only be used in conjunction with a quantitative risk assessment based on clinically validated thresholds for the content of the total food allergen protein in the final food product. Therefore, reliable and comparable data from allergen measurements in food products have to be available.

Currently, the allergen content in food is measured using different analytical measurement principles including immunoassays, PCR and mass spectrometry. These measurement principles target a variety of analytes like epitopes of proteins, DNA fragments or peptide markers. In addition, analytical results are expressed in different properties and measurement units. Consequently, the first step towards achieving a comparability of allergen measurement results is an ‘unambiguous definition of a common measurand’, i.e. of the quantity intended to be measured. According to EU legislation, the unintentional presence of the allergenic ingredient has to be communicated to the consumer in such a way that they will easily realize and understand the threat posed.

The General Food Law requires food to be safe for consumption [2]. This law relies on industry-wide risk assessment procedures. The procedures for food allergens are based on, among others, the most recent clinical threshold data [3, 4]. The majority of such data have been produced by challenging affected patients with known amounts of the offending ‘total food allergen protein’. To be meaningful for assessing food safety, the common measurand is neither the ‘content of the individual proteins’ nor the ‘total food’, but the ‘sum of the total proteins from a particular allergenic food ingredient in a whole food’. This poses somewhat of a dilemma as currently the analytical methods suggested above only detect specific targets. When quantifying these targets, the conversion of measurement results into the common measurand (reported quantity) and the associated uncertainties are often overlooked. This common quantity is the ‘mass of total allergenic ingredient protein per mass of food’ [5] as this is the information on which risk assessments and the establishment of clinical thresholds are based on. Thus, the link between clinical thresholds, risk assessments and analytical measurement results is the common measurand.

Another key element to ensure the comparability between analytical results is their metrological traceability to a common reference. This should preferably be related to the International System of Units (SI) [6]. The practical realization of such traceability requires appropriate calibration systems. As the common measurand for allergen measurements specified above belongs to quantities, which are not directly measurable, the implementation of a reference measurement procedure for the allergenic ingredient would enable the establishment of analytical results traceable to the same reference.

Action levels or ‘thresholds’ can be set to manage allergen risks for which it is estimated that 1% or 5% of the allergic population are likely to elicit a reaction (cf. ED01 or ED05, respectively) [3]. They rely on allergen reference doses, which are proposed based on clinical eliciting doses (VITAL [3], EAACI [7]) and take the consumption portion size into account. In Germany, the Netherlands and Belgium, national expert groups set the action levels [8–10].

According to the RASFF database [11], milk is the main commodity causing allergen-related product withdrawals or recalls in the EU. The product distribution of these recalls showed that cereals and bakery products were the most commonly involved products [5]. Allergy to cow’s milk is one of the most common food allergies in early childhood and it can persist through adult life, forcing the allergic individuals to a complete elimination of milk from their diet. The agents that cause allergic reactions are specific proteins contained in the bovine milk. Major milk allergens include proteins from the casein fraction (alpha-S1-casein, alpha-S2-casein, beta-casein, kappa-casein) and from the serum or whey fraction, namely beta-lactoglobulin, alpha-lactalbumin, bovine lactoferrin and bovine serum albumin [12]. A VITAL 2.0 reference dose of 0.1 mg milk protein (ED01) was previously suggested [3]. In 2019, this dose was set to 0.2 mg/kg as total milk protein in VITAL 3.0 [13]. Assuming a minimum portion size of 200 g, a measurement range of 1 to 10 mg/kg as total milk protein is suggested to support the quantitative risk assessment [14].

The aim of this work was to develop a proof of concept for reference methods for food allergen quantification based on mass spectrometry. The method would aim to quantify the common protein targets used by ELISA, the peptides used in MS as well as the mass fraction of total food allergen protein present. Such methods will be useful for the production of certified reference materials (CRMs), whereby end users can assure the measurement of their analytical target as well as the intended measurand. Moreover, the feasibility of a proof-of-concept study by applying an analytical approach for the determination of total milk protein in a model food baked product was investigated.

## Materials and methods

### Materials

The reagents used for protein extraction and digestion including urea, ammonium bicarbonate (AMB), dithiothreitol (DTT), iodoacetamide (IAA) and trifluoroacetic acid (TFA) were purchased from Sigma (St. Louis, MO, USA). Solvents including ULC grade acetonitrile (ACN), ULC water and 99% formic acid (FA) were purchased from Biosolve (Valkenswaard, the Netherlands). Trypsin Gold-Mass Spec Grade was purchased from Promega (Madison, WI, USA). HyperSep™ 200 mg C18 cartridges were obtained from Thermo Scientific (Biopolymers, Ulm, Germany).

Synthetic peptide analogues with an organic purity greater than 98% were purchased from Thermo Fisher Scientific (MA, USA). The following eleven sequences, representing individual proteins of cow milk, were synthesized: FFVAPFPEVFGK (FFV) and YLGYLEQLLR (YLG) for αS1-casein (CASA1); ALNEINQFYQR (ALN), FALPQYLK (FAL), NAVPITPTLNR (NAV) and VIPYVR (VIP) for αS2-casein (CASA2); AVPYPQR (AVP) and VLPVPQK (VLP) for β-casein (CASB); YIPIQYVLSR (YIP) for κ-casein (CASK); ALPMHIR (ALP) and IPAVFK (IPA) for β-lactoglobulin (LACB). The protein short names in parentheses are the UniProtKB identifiers [15].

Stable isotope-labelled (SIL) peptide analogues of the eleven sequences listed above were purchased from JPT (JPT Peptide Technologies GmbH, Germany) as crude lyophilized peptides with isotopic purities higher than 99%. In these SIL peptides, the C-terminal lysine (K) or arginine (R) were replaced by ^13^C_6_-^15^N_2_-lysin (K*) or ^13^C_6_-^15^N_4_-arginine (R*).

Milk ingredients were purchased from BIOSERVICE Zach GmbH (Austria) as spray dried skimmed milk powder (SMP) (09G010).

### Preparation of peptide standards

All natural peptide stock solutions were prepared gravimetrically by weighing 10 mg of the individual lyophilized peptides. The peptides were dissolved in 10 g of 20% ACN (v/v):80% (w/v) 0.1 M AMB buffer, except for the peptide ALN which was prepared in mass spectrometry grade water only. Peptide stock solutions representing the milk proteins CASA1, CASA2, CASB, CASK and LACB were prepared at 1 and 10 nmol/g from the individual peptide stock solutions by mixing gravimetrically the calculated amount for each peptide. A synthetic milk peptide solution was prepared from the 1 nmol/g protein stock solutions by mixing gravimetrically the calculated amount for each protein according to their natural concentrations theoretically calculated in the SMP ingredient [16].

The peptide purity and sequence were confirmed by analysing the individual peptide stock solutions by mass spectrometry in both full MS scan mode and DDA acquisition mode using a Synapt G2 HDMS Q-TOF-MS (Waters, Manchester, UK) as well as by HPLC-UV (Agilent 1260 Infinity II, RIC, Kortrijk, Belgium). The standard stock solutions were stored at − 20 °C and stability was checked over 6 months. An amino acid analysis (AAA) was performed on the individual peptide stock solution to determine accurately the peptide concentration according to Muñoz et al. [17]. The analysis was performed by quantifying the amino acids alanine, proline, valine, isoleucine, leucine and phenylalanine whenever the peptide sequence allowed it. A minimum of two amino acids were considered for the peptide solution quantification. Since the concentration for the peptide stock solution of YLG could not be determined based on this criterion, the purity was estimated as the average of the purities of all the other peptides. This reasoning is based on the fact that all the peptides were synthesized in parallel. The uncertainty of this purity estimate was regarded as a rectangular distribution with a width equal to the range of the purities. The mass fraction of YLG was calculated from the gravimetric value corrected by the purity estimate. The mass fractions of the other peptides were assigned from the amount-of-substance concentration determined by amino acid analysis.

The individual SIL peptides were re-suspended in 20% ACN (v/v):80% (w/v) 0.1 M AMB buffer. The concentrations were determined by reverse isotope dilution mass spectrometry (IDMS) using the natural peptide analogues. A synthetic milk SIL peptide solution was prepared from the individual peptide stock solutions in order to obtain a natural/SIL peptide peak area ratio of 1 at the mass fraction of 11.25 mg of protein ingredient per kilogram of cookie.

### Calibration standard solutions

For the calibration standard solutions, working stock solutions were prepared from the synthetic milk peptide stock mixture at mass fractions representative of 2.5, 6.25, 10, 13.75, 17.5, 21.25 and 25 mg total ingredient protein per kilogram cookie. The concentrations of the peptides in 0.1 M AMB solution were in the range of 0.45 to 20.63 pmol/g. The lower concentration of 0.45 pmol/g corresponds to a CASA2 protein concentration, the lowest abundant protein level in the lowest standard, of 2.5 mg/kg, whilst the higher concentration of 20.63 pmol/g corresponds to the CASA1 protein concentration, the highest abundant protein level in the highest standard of 25 mg/kg. The same peptide mixture for the SIL analogues was prepared at the mass fraction of 11.25 mg/kg, which corresponds to the midpoint of the calibration curve. In this case, an equal amount of SIL working solution was added either to standards or samples.

### Incurred cookie preparation

Model cookie materials were prepared incurred by adding the different amounts of milk protein ingredient before baking. The skimmed milk powder (SMP) used contained 33.86% (w/w) protein. This was obtained by applying a conversion factor of 5.85 [18, 19] to the nitrogen content as determined by the Dumas method, which was performed on the raw ingredient. The total protein content of the SMP was used to calculate the incurred allergen protein mass fraction. This serves only as an estimate for the nominal values without any associated uncertainties.

The individual batches of cookies were prepared by mixing sugar, dairy-free margarine, wheat flour, raising agents and the appropriate amount of SMP ingredient. Cookies were baked at 160 °C for 14 min, ground in a kitchen blender and then re-ground under liquid nitrogen with an ultra-turrax (IKA, ULTRA-TURRAX T10 Basic, China). Finally, the fine powder material was vacuum-packed and stored at − 20 °C until further use. Materials were prepared with protein nominal mass fractions of 2.3, 4.6, 9.2 and 18.4 mg/kg in the wet dough, which correspond to 2.7, 5.3, 10.7 and 21.3 mg/kg in baked cookie. Furthermore, a blank cookie was prepared with an identical composition without the addition of the SMP ingredient. The baked cookie showed a mass (moisture) loss of 16% when comparing the mass of the wet dough to the mass of the baked cookie.

The milk content in all food ingredients used in the preparation of the cookies was below 0.3 mg/kg as total milk when measured with ELISA test kits for LACB and casein proteins (Morinaga Institute of Biological Science Inc., Yokohama, Japan). The homogeneity of incurred cookies was tested for all preparation levels using the same ELISA test kits: two independent extractions were performed from six different samples (with a minimum sample intake of 1 g) and analysed in triplicate. The relative standard deviations were less than 3%, indicating that the homogeneity with respect to the milk protein content of all the cookie materials was fit for the purpose of this study [20].

### Protein extraction and digestion

The proteins from 1 g of ground cookie were extracted by adding 15 g of extraction buffer (5 M urea, 50 mM AMB, 50 mM DTT, pH 8) at 4 °C. The cookie material was homogenized by two cycles of vortexing during 2 min and sonication during 2 min. All the steps were performed keeping samples on ice. Then, the samples were agitated in a vertical rotator for 3 h at 4 °C. The homogenate was centrifuged at 9500 rpm, corresponding to an RCF at 9383*g*, during 45 min at 4 °C. Afterwards, the supernatant was carefully collected between the bottom precipitate and the upper fatty layer. All sample preparations from this point were performed using 2-ml Protein LoBind microcentrifuge tubes (Eppendorf AG, Hamburg, Germany). Protein extract samples, calibrants and SIL standard solutions were gravimetrically added. An aliquot of 0.25 g of the extracted protein solution was used for protein digestion. For protein reduction, 50 μl of 80 mM of DTT in 25 mM AMB was added and the samples were incubated at 37 °C during 45 min. Cysteine residues were then blocked by the addition of 50 μl of 450 mM iodoacetamide in 25 mM AMB and the resulting solution was incubated at room temperature for 1 h in the dark. Then, 0.25 g of the natural (Nat) peptide mixture solution was added to the blank cookie extract to prepare the Nat-QC samples and the calibrants. Similarly, 0.14 g of labelled peptide mixture (SIL, used as internal standard) solution was added to the blank cookie extract to prepare the SIL-QC sample, the calibrants and all other samples. A final set of blank samples was prepared without any addition of SIL or Nat peptide mixture solutions. The samples were diluted by addition of 800 μl of digestion buffer (18% (v/v) DMSO in 25 mM DTT). Then, trypsin enzyme was added at a ratio of enzyme to substrate of 1 to 50 [21] and digestion was performed for 14 h at 37 °C. The enzymatic reaction was stopped by adding 5 μl FA. The total protein content (including the wheat flour proteins) was estimated by using the RC DC™ protein assay (reducing agent compatible, Bio-Rad, Hercules, CA, USA) with bovine serum albumin as the calibration standard. The RC DC kit is based on the Lowry protocol. The average protein concentrations determined in the cookie protein extract was used to calculate the appropriate trypsin amount to be added to all the samples investigated.

### Mass spectrometry

Prior to the analysis of the samples by mass spectrometry, the samples were de-salted and concentrated. For achieving that, digested samples were cleaned on 200 mg HyperSep™ C18 columns (Thermo Scientific, Biopolymers, Ulm, Germany). The columns were activated with 2 ml of ACN and equilibrated with 2 ml of 10 mM ammonium acetate in 0.1% TFA (equilibration buffer). After loading the sample, diluted with 2 ml of equilibration buffer, the column was washed with 1 ml of 10 mM ammonium acetate in 0.1% TFA, 2 ml of 0.1% FA and 1 ml of 5% ACN in 0.1% FA. Peptides were first eluted in 1.8 ml of 60% ACN in 0.1% FA, then concentrated using a speed vac centrifuge (Martin Christ, Germany) before dilution with mass spectrometry grade water up to 0.5-ml final volume.

A Xevo TQ-S triple quadrupole mass spectrometer (Waters, Manchester, UK) equipped with a trizaic ion source was used to acquire selected reaction monitoring (SRM) data for this study. Peptide mixtures were separated on a 150 μm × 100 mm Peptide BEH C18 130-Å, 1.7-μm UPLC iKey column with an integrated ESI emitter, which formed part of the ionKey-based separation system (Waters, Manchester, UK). The sample injection volume was 5 μl using a full loop injection. A dual pump reverse flush trapping configuration was applied. The sample was loaded on a trapping column (Symmetry 300 C18, 5 μm, 300 μm × 50 mm MClass) and an isocratic flow of 100% A for 2 min with a flow rate of 10 μl/min was applied. The trapping column was then back-flushed in line with the iKey column at a flow of 2 μl/min utilizing the following gradient: 0–5% B for 0.5 min, 5–25% B for 17.5 min, 25–40% B for 4.5 min. Solvent A was 0.1% FA in water and solvent B was 0.1% FA in ACN. Each sample run was followed by an injection of 5 μl of ACN applying a gradient of 0–100% B for 3 min, 100–0% B for 1 min and kept at 100% A for 4 min with a flow rate of 10 μl/min in order to condition and equilibrate both the trap and analytical columns between sample injections. The collision energies (CE) and cone voltages (V) applied whilst performing the individual SRM experiments were experimentally obtained using the synthetic natural peptides in neat solution (Table 1). The capillary voltage was set to 3.2 kV, the collision gas to 0.1 ml/min and the cone voltage to 25 V. SRM transitions were scheduled along the acquired time using a fixed dwell time of 40 ms and a minimum of 15 points across the chromatographic peak. Two transitions for the natural peptides and one transition for the labelled peptides were acquired. The quadrupole parameters were set to unit mass resolution for Q1 and Q3. All sample extracts were assayed in triplicate. After each injection, the autosampler needle was rinsed with a strong washing solvent (2% DMSO in 95% ACN:0.1% FA in water) followed by a weak washing solvent (1% ACN:0.1% FA in water) to minimize carry-over effects. A system suitability test (SST), including full scans and daughter ion scans, was performed before each set of analysis to verify that the method performed within its specifications (retention time, *m*/*z* of precursor and products). Three quality control (QC) samples were prepared, called BLK-QC containing the blank matrix, Nat-QC containing the blank matrix spiked with the midpoint calibrant and SIL-QC consisting of a blank matrix spiked with the internal standard labelled peptide blend. The instrument analysis run order was divided into 5 blocks. The first and last block contained QC and calibrant samples analysed starting with the highest calibrant concentration point 7, followed by point 0 (SIL-QC) and then calibrant points 1–6 in increasing concentration order. Three consecutive blocks were analysed containing all the samples of the experiment and the calibration curve (points 0 to 7) in a randomized order.

**Table 1:** Specifications for the selected reaction monitoring (SRM) acquisition by LC-MS/MS of unlabelled and labelled (*) peptides. The protein short names in parentheses are the UniProtKB identifiers [15]

Protein	Peptide sequence	Retention time min)	Precursor monoisotopic mass (MH^+^)	Transition (m/z)	Collision energy (eV)	Cone voltage (V)
CASA1	FFVAPFPEVFGK	19.6	1383.746	693.0→465.2 (b4)	14	25
	(FFV)			693.0→920.4 (y8)	18	25
			(*) 1391.746	697.0→928.4 (y8)	18	25
	YLGYLEQLLR	18	1267.704	634.5→771.3 (y6)	16	25
	(YLG)			634.5→991.3 (y8)	20	25
			(*) 1277.704	639.5→1001.3 (y8)	20	25
CASA2	ALNEINQFYQK	10.4	1366.688	684.4→438.1 (b4)	20	15
	(ALN)			684.4→827.4 (y6)	20	15
			(*) 1374.688	688.4→835.4 (y6)	20	15
	FALPQYLK	13	979.561	490.3→648.3 (y5)	14	15
	(FAL)			490.3→761.3 (y6)	14	15
			(*) 987.561	494.3→656.3 (y5)	14	15
	NAVPITPTLNR	9.6	1195.679	598.3→701.4 (y6)	20	15
	(NAV)			598.3→911.4 (y8)	15	15
			(*) 1205.679	603.3→921.4 (y8)	15	15
	VIPYVPR	7.4	746.455	373.8→437.4 (y3)	15	15
	(VIP)			373.8→534.4 (y4)	15	15
			(*) 756.455	378.7→544.4 (y4)	15	15
CASB	AVPYPQR	4.7	830.452	415.8→400.2 (y3)	15	20
	(AVP)			415.8→660.3 (y5)	15	20
			(*) 838.452	420.8→670.3 (y5)	15	20
	VLPVPQK	6	779.49	390.8→372.2 (y3)	15	20
	(VLP)			390.8→568.2 (y5)	10	20
			(*) 787.49	394.8→380.2 (y3)	15	20
CASK	YIPIQYVLSR	15.1	1251.71	626.5→488.2 (b4)	20	25
	(YIP)			626.5→765.2 (y6)	20	25
			(*) 1259.71	631.5→493.2 (b4)	20	25
LACB	ALPMHIR	6.6	837.476	419.8→425.3 (y3)	15	26
	(ALP)			419.8→653.5 (y5)	15	26
			(*) 837.476	424.8→663.5 (y5)	15	25
	IPAVFK	8.1	674.424	337.8→393.2 (y3)	15	25
	(IPA)			337.8→464.2 (y4)	15	25
			(*) 682.424	341.8→472.2 (y4)	15	15

### Data analysis

Mass spectrometric data analysis was performed using the TargetLynx software associated with MassLynx version 4.1 (Waters). The chromatographic peaks were integrated automatically using the following parameters for peak integration: a mean smoothing method with 2 smoothing iterations and a smoothing width of 5 points, 10,000 peak threshold for shoulder detection and 100 peak absolute area. For each natural and isotopically labelled peptide pair, the peak area ratio natural/IS peptide was calculated. The data evaluation was performed using Microsoft Excel and the Analyse-it method validation edition software (Analyse-it Software, Leeds, UK) [22].

The amount of each signature peptide in each sample was calculated via the following equation:

1 $$b\left({\mathrm{pep}}_{i,j,k}\right)=\frac{R_{i,j,k}-{\beta}_{0,i,j}}{\beta_{1,i,j}}$$

where

*b*(pep_*i,j,k*_) is the molality of the *i*th signature peptide of the *j*th marker protein in the *k*th unknown sample in [mol/g];

*R*_*i,j,k*_ is the measured isotope ratio of signal of *i*th signature peptide over signal of its SIL analogue of the *j*th marker protein in the *k*th unknown sample;

*β*_*0,i,j*_ is the intercept of iteratively reweighted least square regression model for the *i*th signature peptide of the *j*th marker protein; and

*β*_*1,i,j*_ is the slope of iteratively reweighted least square regression model in [g mol^−1^] of the *i*th signature peptide of the *j*th marker protein.

The mass fraction of the *j*th marker protein in the *k*th test solution is then calculated as follows:

2 $${w}_{j,k}=b\left({\mathrm{pep}}_{i,j,k}\right)\frac{m_{\mathrm{Exsolv},k}}{m_{X,k} * {m}_{\mathrm{Extr},k}}{M}_j\ast {f}_{\mathrm{ext}}\ast {f}_{\mathrm{dig}}\ast {f}_{\mathrm{purity}}$$

where

*w*_*j,k*_ is the mass fraction of the *j*th marker protein in the *k*th unknown sample;

*b*(pep_*i,j,k*_) is the molality of the *i*th signature peptide of the *j*th marker protein in the *k*th unknown sample in [mol/g];

*m*_Exsolv*,k*_ is the mass of the extraction solvent added to the *k*th unknown sample;

*m*_*X,k*_ is the mass of the test portion in the *k*th unknown sample;

*m*_Extr*,k*_ is the mass of the extract aliquot used for the *k*th unknown sample;

*M*_*j*_ is the average molar mass of the *j*th marker protein accounting for natural variation of the amino acid sequence;

*f*_ext_ unity factor bearing the uncertainty contribution due to extraction;

*f*_dig_ unity factor bearing the uncertainty contribution due to digestion; and

*f*_purity_ unity factor bearing the uncertainty contribution due to purity.

Once the mass fractions of the marker proteins were known, the mass fraction of the total content of milk protein (TCMP) in an unknown sample could be calculated as:

3 $${w}_{\mathrm{TCMP},k}={w}_{j,k}\ast {\mathrm{CF}}_j$$

where

*w*_TCMP,*k*_ is the mass fraction of TCMP in the *k*th unknown sample and

CF_*j*_ is the conversion factor accounting for the contribution of the *j*th marker protein to the total cow’s milk protein.

Each of the signature peptides is present at 1 mol per 1 mol marker protein. A complete digestion is evidenced by equimolarity of all respective peptides per marker protein [21]. The standard error of the mean of all signature peptides belonging to one marker protein is used as the between-peptide uncertainty. All results reported in this manuscript are expressed in milligrams per kilogram as total milk protein related to baked cookies.

## Results and discussion

**Fig. 1:**
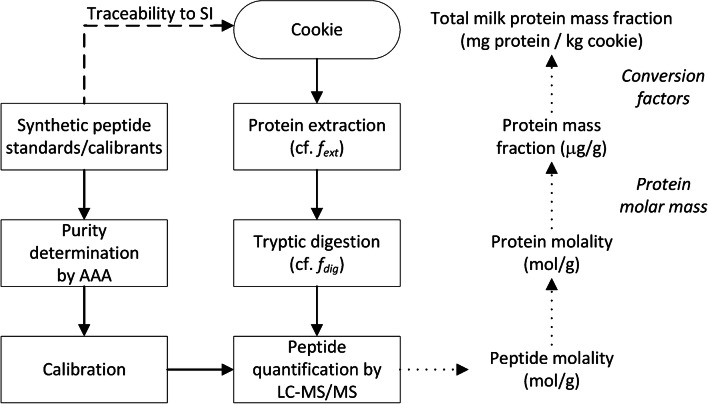
Workflow for the determination of the total milk protein content in baked cookies (expressed in mg protein/kg cookie) derived from the quantification of the signature peptide markers for the cow’s milk allergenic proteins

Designing a reference measurement method for quantifying food allergens in a complex matrix requires careful considerations with respect to metrological traceability throughout the entire analytical process. The goal of the development reported here was an MS-based method, which should provide data that can be expressed in the required common reporting parameter, i.e. milligrams of total milk protein per kilogram of cookie, with traceability to the SI. Since no analytical method quantifies directly total milk protein in a complex sample, such as the cookie employed as a processed food model here, a combination of measurement steps for surrogates of the measurand and mathematical transformations of measured amounts into other property values had to be developed. The MS method described here quantifies the amount concentration of specific signature peptides from allergenic milk protein markers in the cookie. As only one copy of each peptide is present in each protein [21], the amount concentration for the respective peptides from the same marker protein should be equimolar and convey the amount concentration of the parent marker protein [23]. However, a conversion of the amounts from the individual markers to the amount of total milk protein is necessary. Figure 1 depicts the complete analytical approach developed. The workflow initiates with the preparation of the sample, followed by the extraction of the proteins from the cookie. A tryptic digestion of the proteins contained in the extract is performed to produce the signature peptides, which afterwards are analysed by a targeted LC-MS/MS step. The metrological traceability of the final result to the SI is ultimately ensured via synthetic peptides used as calibrants for the signature peptides, which have been value-assigned by AAA. A complete digestion and equimolar release from their origin proteins is assumed. The determination of each signature peptide allows the accurate quantification of the individual marker proteins and by applying conversion factors the consequent determination of the total milk protein content in the starting material. Each step of the analytical procedure is described in the following.

### Selection of the signature peptides

The selection of the peptide markers is based on a previous study, where a list of robust peptide markers for milk ingredients in an incurred material was proposed for the development of a quantitative MS-based method [21]. In the current study, a set of eleven peptide markers from an incurred cookie were measured by SRM at the clinically relevant level of 10 mg milk ingredient protein per kilogram cookie. The final selection of the eleven signature peptides (Table 1) was based on the intensity of the peak areas (S/N > 3). The selected signature peptides are suitable for quantification as they meet the following criteria: (i) being unique to their originating proteins; (ii) being in line with the selection rules established for MS methods [14]; (iii) allowing the preparation of isotopically labelled analogue peptides and (iv) being stable regarding chemical modifications during the processing of the matrix and during the analytical procedures described by Nitride et al. [21].

It should be noted that ALP, containing methionine prone to oxidation, does not follow the guidelines as laid down by Johnson et al. [14]. However, due to the lack of other suitable peptide markers, this major peptide constituent was observed during the digestions, and was later retained for further calculation. Hence, a total of eleven peptides were suitable signature markers used for the quantification of the clinical levels investigated, ranging from 1 to 10 mg/kg [24].

The eleven signature peptides are specific to cattle (*Bos taurus*). Many of them were also found in protein sequences of the water buffalo (*Bubalus bubalis*), except for FAL and VIP from CASA2 and IPA from LACB. Moreover, five peptides (ALN, ALP, VLP, YIP and YLG) were detected via the NCBInr database in protein sequences of sheep (*Ovis aries*) and goat (*Capra hircus*). Therefore, those peptides could potentially also be used to detect milk from other species.

The selected peptides were used to analyse the five most abundant allergenic proteins, namely CASA1 (Bos d 9), CASA2 (Bos d10), CASB (Bos d11), CASK (Bos d12) and LACB (Bos d5). The casein fraction, amounting to about 80% (w/w) of the total milk protein [16], was quantified via four proteins based on the measurement of nine signature peptides. The whey fraction, representing about 20% (w/w) of total milk protein [16], was quantified via LACB by measuring two signature peptides. It has to be kept in mind that LACB represents about 50% of the total content of the whey proteins. In total, the selected peptide and protein markers cover about 90% (w/w) of the total milk protein content.

### Analytical procedure

As indicated in Fig. 1, the sample preparation consists of extraction of the proteins from the material, digestion of the extracted proteins using trypsin as a proteolytic enzyme and clean-up of the digested extract. Afterwards, the released peptides are measured by mass spectrometry.

The most critical steps during sample preparation for any MS-based measurement are the extraction and digestion steps. Their conditions were investigated and optimized by Nitride et al. [21] applying a statistical experimental design. That study aimed at achieving the optimal extraction yield for each individual protein. The complete digestion was ensured and an equimolar release of the signature peptides was obtained. Since no universal preferential extraction and digestion conditions were found in this previous study [21], resulting in the complete extraction of every protein, the selected conditions for extraction and digestion were a compromise of the optimal conditions for each peptide and protein investigated covering a comprehensive range of the milk allergenic protein fraction. The variance of the extraction was assessed during the method validation. The variability of the extraction from the five targeted milk allergenic proteins was within the uncertainty of the measurement results.

### Peptide calibrants

The quality of the peptides employed as calibrants and labelled peptide standards (SIL) is crucial. The unlabelled and labelled standard peptides were analysed by LC-MS/MS to confirm the correct peptide sequences. The unlabelled peptide standards were also measured by HPLC-UV to check for potential impurities that may interfere with the AAA (data not shown). No major peptide impurities were found and the peptide purities were determined to be above 98% (w/w) in agreement with the specifications given by the peptide provider*.* The solutions of the unlabelled peptides were quantified by AAA and large deviations were found compared with the gravimetrically prepared solutions nominally containing 1 mg/g. The content determined by AAA differed by 21% to 56% from the expected mass fraction of the peptide standard solutions (see Electronic Supplementary Material (ESM) Table S1). Although the peptide sequence impurities as determined by MS and HPLC-UV were less than 2%, chemical impurities such as trifluoroacetic salts and the moisture content of the lyophilized powder peptides may have contributed to such large discrepancies to the manufacturer’s assigned purity of the lyophilized peptides.

The results obtained demonstrate the importance of correctly determining the amount-of-substance concentration of the peptide standard solutions. These solutions are used as calibrants and thus form the basis for the metrological traceability of the food allergen measurement results. The use of peptide standards, value-assigned by AAA, for obtaining SI-traceable values for MS measurement results on completely digested proteins has been discussed in the literature [25, 26].

Milk proteins are present at different mass fractions. Thus, the composition of the milk in nature was a factor included in the preparation of the calibration curves (the theoretical composition is outlined in the ‘Molecular masses and conversion factors to total milk protein’ section). Therefore, each surrogate peptide standard calibrant is present at the natural abundance of each milk protein. A blend of the value-assigned standard peptide stock solutions was gravimetrically prepared to mimic the milk composition. The concentrations of the five milk protein markers were obtained from the individual peptide standards according to the theoretical composition of the cow’s milk protein content. This synthetic milk standard solution of peptides was used to prepare seven equidistant mass fraction calibrants ranging from 2.5 to 25 mg total milk protein per kilogram of cookie.

**Fig. 2:**
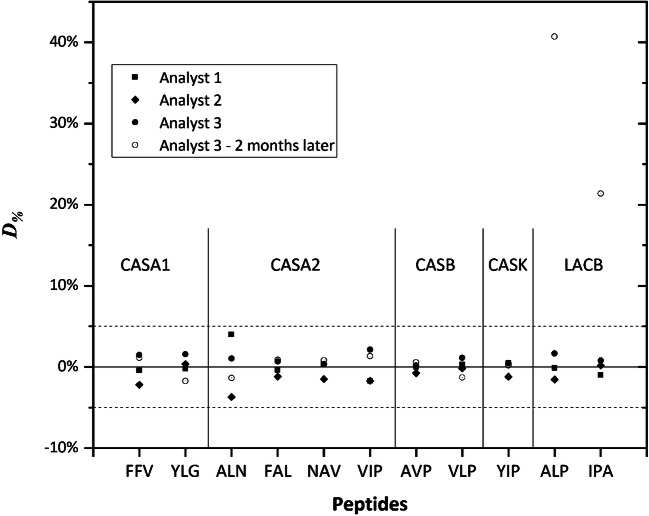
Assessments of the variability for the batch-to-batch peptide standard blend preparation. Percent differences (*D*_%_) calculated for the individual peptide stock solutions analysed by three analysts

To study the variability associated with the preparation and stability of the peptide standard blend, three independent preparations were made by three analysts as well as another independent preparation after 2 months by one of the analysts. A percent difference (*D*_%_) is calculated as the difference for each peptide and analysis between the amount of each peptide measured by IDMS and their nominal amount in picomole per gram. Figure 2 shows relative differences in the standard blend preparation well below 5%. Larger *D*_%_ values are only observed for the two peptide markers of LACB (ALP and IPA) when the peptide blend solution was prepared after 2 months. This may indicate that these two peptide standards might be less stable in solution.

### Traceability of the measurement results

According to the International Vocabulary of Metrology (VIM 3, [27]), metrological traceability is defined as the ‘property of a measurement result whereby the result can be related to a reference through a documented unbroken chain of calibrations, each contributing to the measurement uncertainty’. Achieving metrological traceability to the SI for results on the mass fraction of ‘total milk protein’ is far from trivial. The first challenge relates to the unambiguous definition of the measurand: ‘total amount of milk protein in baked cookie’ expressed in ‘mg total milk protein per kg cookie’, which derives from the experimental quantification of the amount of every individual targeted milk protein. The second challenge relies on the establishment of the calibration chains required for the quantification of non-directly measurable targets, namely peptides and protein molecules in a complex matrix, such as baked cookie.

The latter challenge seems to be equivalent to the task of obtaining SI-traceable measurement results of a protein used as a biomarker in clinical diagnostics [28]. The first challenge, however, introduces additional levels of variability into the design and realization of the traceability chains (actually ‘traceability nets’). The sum parameter ‘total milk protein’ requires information on the identity and mass fractions of several proteins in the sample. However, the protein structures and relative composition for milk have a biological variability between different animal species and even between similar individuals. This ‘definitional uncertainty’ of the measurand [27] has to be taken into account when determining the food allergen content in a sample. Therefore, the fit-for-purpose approach developed in the study described here makes use of conversion factors to transform quantification data from MS-measurable markers (molecules or molecular fragments) to the final measurand.

The established traceability chain can be understood by following the analytical workflow (Fig. 1) in the opposite direction. The final measurement result, i.e. the mass fraction of total milk protein in cookie, is related to the obtained mass fractions of individual milk proteins. Conversion factors for the contribution of each target protein to the composition of total cow’s milk protein are required in this calculation. The protein mass fractions are based on the determined amounts of substance for each target milk protein in a given sample mass of cookie. For this mathematical operation, the uncertainties of the natural mass fractions and the molar masses for the individual proteins have to be considered. The values for ‘mol (target) protein/g cookie’ are directly related to the equivalent peptide amounts assuming that one mol peptide relates to one mol protein. The data for the signature peptide amounts originate from the evaluation of the MS peptide signals measured on the digested protein extract obtained from the cookie sample. The traceability of the MS signals has been established by relating them via dedicated calibration curves to amounts of synthetic peptides. The purity of these calibration standards had been characterized by AAA and other methods (see the ‘Preparation of peptide standards’ section). Thus, the peptide standards are serving here as ultimate chemical realization of the SI unit mole.

The step-by-step linking of the final measurement result to the SI developed contains a number of ‘less knowns’, which have to be assessed with respect to their influence on the fitness-for-purpose of the approach. Investigations on the extraction and digestion efficiencies have already been reported [21]. Another weaker link in the traceability chain is the purity of the peptide standards. The accuracy of realizing a specified amount of a peptide does depend not only on the accuracy of the AAA measurement result but also on the knowledge about the level of peptide impurities that would interfere with the AAA. A remaining uncertainty about that, despite the purity studies with MS and HPLC-UV, has to be estimated and included into the full uncertainty budget. This strategy was also applied to account for the limitations in the knowledge about the biological variability of the protein composition of the parameter ‘total milk protein’, i.e. the uncertainty of the conversion factors. A more detailed description and discussion of the data evaluation and uncertainty estimation is presented hereafter.

### Quantification of the milk proteins

**Fig. 3:**
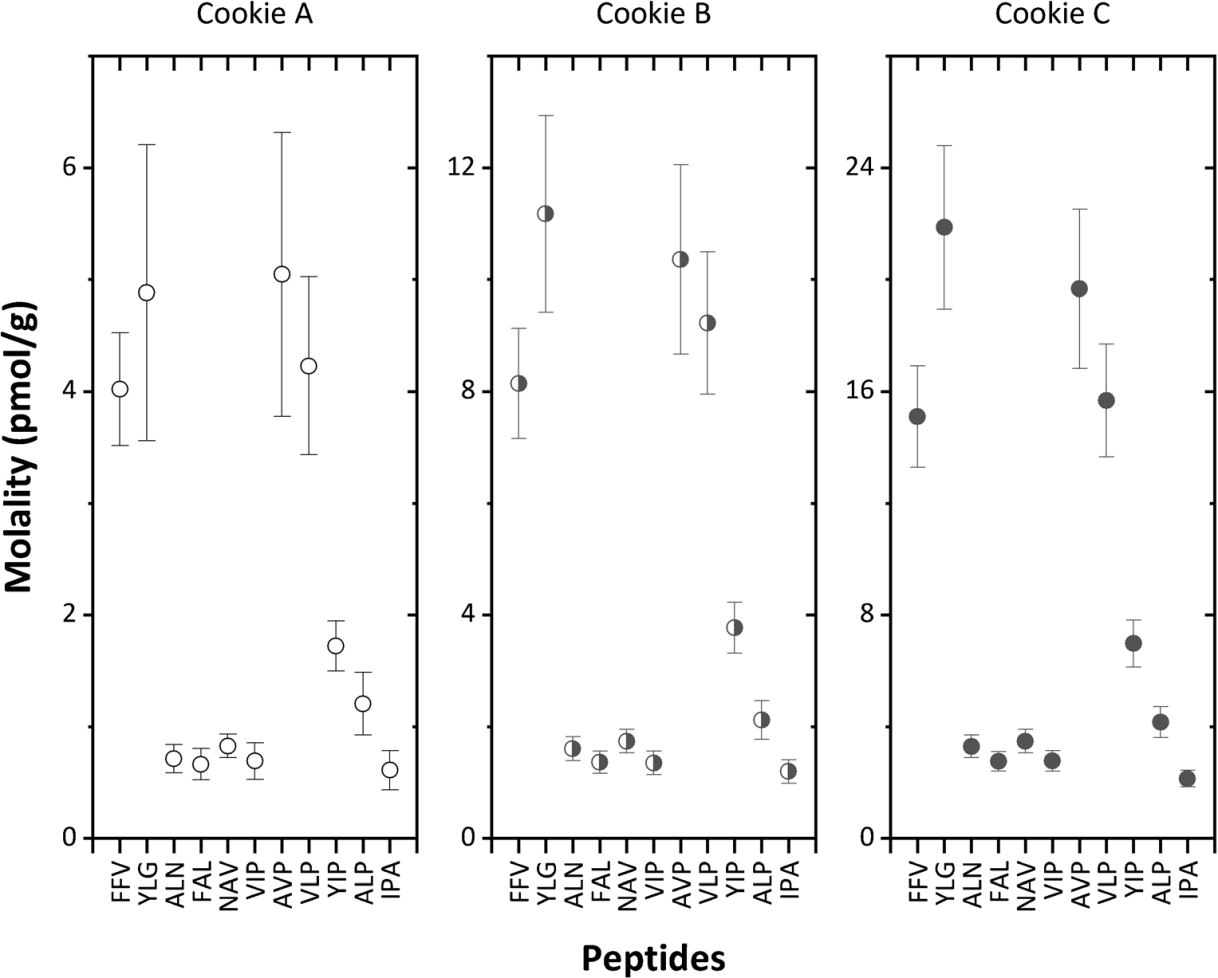
Experimental results (expressed in pmol/g) obtained for the quantification of the eleven signature peptides in three SMP incurred baked cookies (**a**–**c**), with nominal levels of 5.3, 10.7 and 21.3 mg/kg baked cookies, respectively. Expanded measurement uncertainties (with a coverage factor *k* = 2) are presented as error bars

Three cookie samples with nominal total milk protein levels in baked cookies of 5.3, 10.7 and 21.3 mg/kg were analysed. For each sample, three replicates from two digested extracts were measured by SRM. The quantification was based on matrix-matched calibration curves using the value-assigned peptide standards. Figure 3 presents the quantification results for each signature peptide, calculated applying Eq. 1. The following relative standard uncertainties were found when analysing the three cookies: around 5% for FAL, FFV and NAV; around 10% for ALP, AVP, YIP and YLG; and above 20% for IPA and VLP, due to a large scatter of data in the respective calibration curves. A significantly decreasing uncertainty (from ca. 25 to 10%) with increasing incurred total milk protein levels was observed for ALN and VIP.

**Fig. 4:**
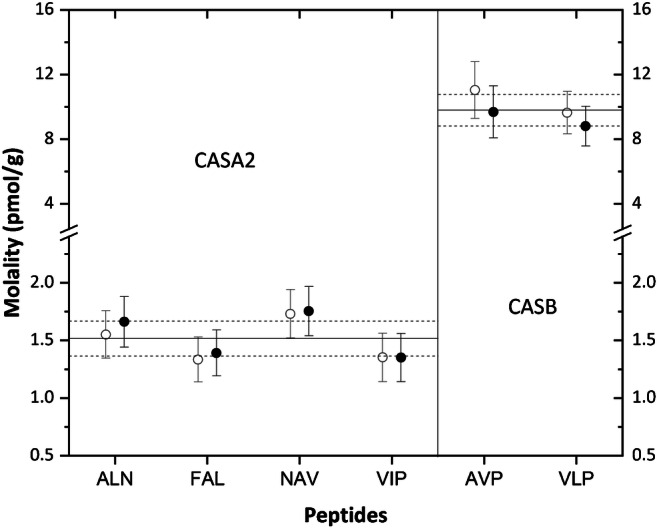
Peptide-to-peptide variability assessment for CASA2 and CASB. Two independent sample intakes were processed (filled and open circles). The experimental results (expressed in pmol/g) are shown for the six marker peptides. Error bars represent expanded uncertainties (coverage factor *k* = 2), whilst the solid lines are the mean protein contents derived from the respective peptide markers. All peptide contents are within 10% of the respective average values (dashed lines)

The mass fraction of each protein was derived from the measured peptide amounts using Eq. 2. Assuming an equimolar release of the signature peptides from their original proteins, the peptide-to-peptide variability was calculated as the relative standard deviation of the molalities for the set of peptide markers of a given protein. This could not apply to the CASA1 protein due to the lack of an assigned value for the YLG standard peptide, nor to CASK characterized by the YIP peptide only. RSDs ranging from 13 to 19% were found for CASA2 and CASB, which is in good agreement with the measurement uncertainties estimated. The large scatter observed for LACB (RSDs of 35–42%) indicates an incomplete digestion of the corresponding peptides (ALP and IPA). Nevertheless, complete digestion was assumed based on the equimolar release observed for CASA2 and CASB, for which duplicate analyses provided overlapping results, as shown in Fig. 4. This was further confirmed by the peptide evolution curves reaching the plateau, previously investigated during the optimization study [21].

### Molecular masses and conversion factors to total milk protein

Molecular masses of each protein were based on sequences of proteins retrieved from the NCBI database for the *Bos taurus* species. They were calculated using the ‘Compute pI/Mw’ tool in Expasy [29]. A total of 25, 5, 14, 8 and 70 sequences were retrieved for CASA1, CASA2, CASB, CASK and LACB, respectively. Ranges and mean values for each protein were derived from the minima and maxima identified (Table 2). All average protein molecular masses of the mature protein form were calculated using the identified sequences.

**Table 2:** Molar mass, protein contents in milk and the corresponding conversion factors for the five proteins of interest. Mean values, the associated standard uncertainties (*u*) and the corresponding relative standard uncertainties (*u*_rel_) are derived from ranges reported in the literature [16, 30]. The conversion factors for the individual proteins are calculated according to Eq. 4, whilst the combined CF(Σ_CAS_) and CF(Σ_prot_) are calculated as the sum of the relevant CF

	Protein	Min	Max	Range	Mean	*u* (*k* = 1)	*u*_rel_
Protein molar mass (g/mol)	CASA1	18629	22975	4346	20802	1255	6 %
	CASA2	23322	24349	1027	23836	296	1 %
	CASB	22641	23623	982	23132	283	1 %
	CASK	18858	18974	116	18916	33	0.2 %
	LACB	18168	18375	207	18272	60	0.3 %
Theoretical protein concentration in milk, *c*_prot_ (g/l)	CASA1	10	15	5	12.5	1.4	12 %
	CASA2	3	4	1	3.5	0.3	8 %
	CASB	9	11	2	10.0	0.6	6 %
	CASK	3	4	1	3.5	0.3	8 %
	LACB	3	4	1	3.5	0.3	8 %
Conversion Factor (CF)	CASA1				0.366	0.046	13 %
	CASA2				0.102	0.010	10 %
	CASB				0.293	0.023	8 %
	CASK				0.102	0.010	10 %
	LACB				0.102	0.010	10 %
	Σ_CAS_				0.864	0.055	6 %
	Σ_prot_				0.966	0.055	6 %

Conversion factors (CF) for each protein are needed to convert quantitative results of the individual marker proteins to the total milk protein. These conversion factors are based on the protein abundances reported by EFSA [16] and Bar et al. [30]. Only recent and up-to-date data were used in the frame of this study for the proof of the developed concept. However, further research is required to derive a harmonized set of conversion factors for milk proteins to be systematically implemented.

The conversion factors, expressed as relative mass contribution of an individual protein to the total milk protein mass (Table 2), are calculated as follows:

4 $$\mathrm{CF}=\left(\frac{c_{\mathrm{prot}}}{w_{\mathrm{MP}}}\right)/{d}_{\mathrm{milk}}$$

where

*d*_milk_ is the milk density of 1.035 kg/l [16];

*w*_MP_ is the total content of milk protein in milk of 33 ± 3 (*k* = 2) g/kg [23]; and

*c*_prot_ is the theoretical individual protein concentration in milk (g/l).

### Uncertainty estimation

Standard uncertainties for the molar mass of the marker proteins were calculated considering the variability of the protein sequences retrieved in NCBI under the individual protein names for *Bos taurus*. Similarly, the standard uncertainties of the individual protein concentrations for milk were calculated considering the natural variability of cow milk. Ranges were calculated as the difference between the maximum and the minimum values. Assuming a rectangular distribution, the corresponding standard uncertainties were calculated as half-ranges divided by$\sqrt{3}$(Table 2) [31].

Overall standard uncertainties were estimated combining the uncertainty contributions of the various sources of variability of the analytical method (input quantities), in accordance with the GUM [32]. The following input quantities were considered: (i) amount of the signature peptides; (ii) purity of the standard peptides; (iii) masses of test material, extraction buffer and aliquot taken for digestion; (iv) molar masses of the individual proteins; (v) conversion factors; and (vi) the extraction and digestion efficiencies. In addition, the standard deviation of the average amount of each peptide per protein is taken into account as a contribution of the incomplete proteolysis.

The law of error propagation was applied to estimate the uncertainties of the measurement results using the Kragten spreadsheet approach [33] further described in the Eurachem guide ‘Quantifying uncertainty in analytical measurement’ [31]. Table 3 presents the experimental results and the corresponding expanded uncertainties obtained for the test material with a nominal value of 10.7 mg TCMP/kg baked cookie. The indexes represent the percent contribution of the main input quantities to the combined uncertainty of the measurement results of the eleven signature peptides. Most of the peptides are determined with a satisfactory combined uncertainty (*k* = 1) ranging from 10 to 16%. In general, the conversion factors (CF) and the molality of the peptide (*b*_pep_) are the main contributors to the combined uncertainty (highlighted in bold in Table 3).

**Table 3:** Results for the mass fractions of total cow milk protein (*w*_TCMP_) obtained from the eleven peptides investigated, the corresponding expanded uncertainties (*U*, *k* = 2) and relative standard uncertainties (*u*_rel_, *k* = 1). The contributions of the main input quantities were investigated: purity of the peptide standard (*f*_purity_); molality of the peptide (*b*_pep_); conversion factor (CF); molar mass of the marker protein (*M*_*j*_); extraction (*f*_ext_) and digestion (*f*_dig_). The ‘indexes’ represent the percent contribution to the expanded uncertainty; major contributors (> 15%) are highlighted in bold

	*w*_TCMP_ (mg/kg)	*U* (*k* = 2) (mg/kg)	*u*_rel_ (*k* = 1)	Indexes					
				*f*_purity_	*b*_pep_	*M*_*j*_	*f*_ext_	*f*_dig_	CF
FFV	6.81	2.01	15%	0.6%	0.4%	**17%**	10%	0.0%	**72%**
YLG	9.41	2.88	15%	3.8%	11%	**16%**	2.1%	0.0%	**67%**
ALN	5.41	1.26	12%	4.7%	6.1%	1.1%	**17%**	0.5%	**71%**
FAL	4.66	1.18	13%	1.2%	11%	1.0%	**28%**	0.0%	**60%**
NAV	6.04	1.33	11%	1.6%	0.8%	1.3%	**17%**	0.0%	**79%**
VIP	4.72	1.17	12%	0.8%	**16%**	1.0%	**20%**	0.0%	**62%**
AVP	13.0	3.0	12%	1.2%	**20%**	1.1%	**29%**	2.7%	**46%**
VLP	11.4	2.3	10%	1.7%	10%	1.5%	**17%**	7.9%	**62%**
YIP	10.7	2.5	12%	2.7%	0.7%	0.0%	**28%**	0.0%	**68%**
ALP	5.43	1.38	13%	2.0%	**21%**	0.1%	9.4%	8.5%	**59%**
IPA	3.17	0.78	12%	0.8%	**28%**	0.1%	5.1%	3.2%	**63%**

### Quantification of the total milk protein content

**Fig. 5:**
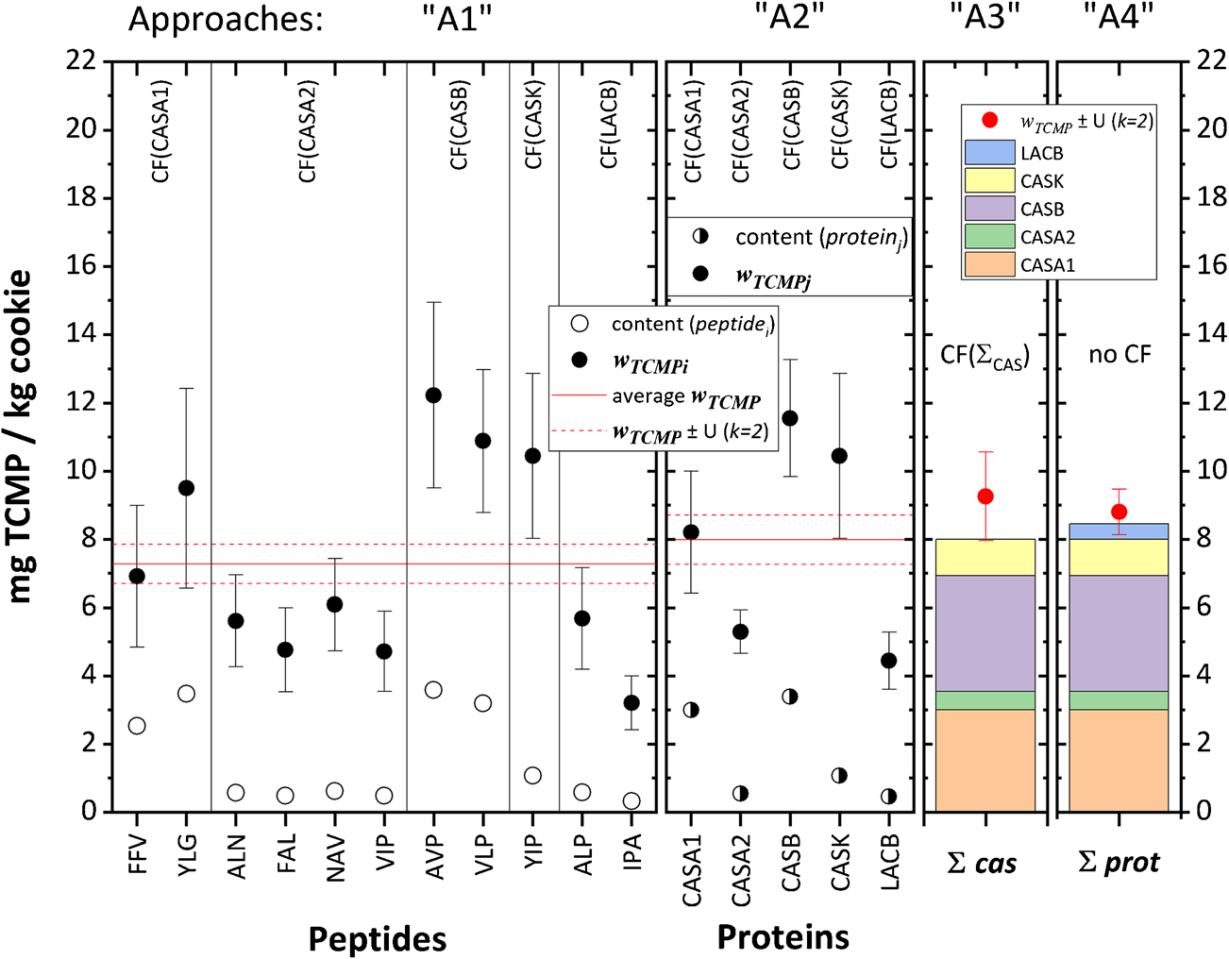
Four approaches for the determination of the content of total cow milk protein (TCMP) in baked cookies (expressed in mg/kg), based on the quantification of 11 peptides (empty circles) further converted in content of TCMP_i_ (approach 1); the quantification of five proteins (half-filled circles) further converted in content of TCMP_j_ (A2); the sum of the four quantified casein proteins (stack) further converted to *w*_TCMP_ (A3) and the sum of the five determined proteins (stack) and the residual protein content of ‘4%’ (A4, for which no conversion factor was applied). The conversion factors used are indicated. TCMP contents are denoted by ‘full circles’, whilst error bars represent ‘expanded uncertainties’ (*k* = 2). The red horizontal dashed lines delimit the expanded ranges *w*_TCMP_ ± *U* (*k* = 2) bracketing the average TCMP contents (red solid lines) calculated using approaches 1 and 2

Four different approaches can be used to derive the TCMP from the quantified amounts of the eleven peptides investigated, using different paths: starting from peptides directly, or via the characterized proteins, or using different fractions of proteins. Each of these approaches leads to different estimates of the measurement uncertainty, based on the calculation path applied. Figure 5 compares the outcome of the four approaches described hereafter, based on the experimental results obtained for the test material with a nominal value of 10.7 mg TCMP/kg baked cookie. The law of error propagation was systematically applied to derive the combined uncertainties.

In approach 1 (A1), each peptide is considered as independent measured marker. The quantified amount for each signature peptide ‘*i*’ is directly converted into the mass fraction of TCMP_i_, using the conversion factor characteristic of the corresponding protein (e.g. CF(FFV) = CF(CASA1) = 0.366, Table 2). Hence, the average of the eleven TCMP_i_ mass fraction values and the associated expanded uncertainty are *w*_TCMP_ (A1) = 7.27 ± 0.57 (*k* = 2) mg TCMP/kg baked cookie.

In approach 2 (A2), each protein is considered as the independently measured marker. At first, the mass fractions of the five marker proteins ‘*j*’ are calculated as the average of the corresponding peptide mass fractions (e.g. CASA1 derived from FFV and YLG). The five protein contents are then converted into TCMP_j_ mass fractions applying the respective CF (Table 2). Hence, the average of the five TCMP_j_ mass fraction values and the associated expanded uncertainty are *w*_TCMP_ (A2) = 7.99 ± 0.73 (*k* = 2) mg TCMP/kg baked cookie.

In approach 3 (A3), *w*_TCMP_ is derived from the sum of mass fractions of four caseins investigated (CASA1, CASA2, CASB and CASK). As in A2, nine peptides allow the quantification of four average casein mass fractions. The sum of these mass fractions is converted into *w*_TCMP_ applying a combined conversion factor (CF_Σcas_ = CF(CASA1) + CF(CASA2) + CF(CASB) + CF(CASK), Table 2). This results in a TCMP mass fraction value and associated expanded uncertainty of *w*_TCMP_ (A3) = 9.26 ± 1.30 (*k* = 2) mg TCMP/kg baked cookie.

In approach 4 (A4), *w*_TCMP_ is derived from the five proteins investigated. The sum of the average mass fractions of these proteins is equal to 8.45 mg/kg with an expanded uncertainty (*U*, *k* = 2) of 0.53 mg/kg, which represents ca. 96% of the total milk protein (CF(Σ_prot_) = 0.966, Table 2). The remaining ‘4%’ (or 0.35 mg/kg) is considered as the half-range of a rectangular distribution, equivalent to a standard uncertainty of 2.3% (= 4% / $\sqrt{3}$, or 0.20 mg/kg). No further conversion factors are required. The remaining ‘4%’ is added to the sum of determined protein mass fractions, to result in a TCMP mass fraction value and associated expanded uncertainty of *w*_TCMP_ (A4) = 8.80 ± 0.67 (*k* = 2) mg TCMP/kg baked cookie. A smaller expanded uncertainty is obtained because it does not bare the 6% contribution of the sum of the conversion factors CF(Σ_prot_) (Table 2).

When choosing a method for the characterization of a reference material, one would normally select the approach with the smallest estimated measurement uncertainty. However, the approach outlined in A4 only relies on one conversion factor (that of the mass fraction of the remaining unmeasured protein contained in milk) and therefore is representative of the actual SMP ingredient used. We are aware that this approach is limited to its use for the determination of total milk protein as the variability of protein sequences and their relative mass fractions is currently not available for other allergenic ingredients. The other approaches may be assessed on a ‘fitness for purposes’ basis for instance, where protein fractions (e.g. whey) have been used as the ingredient.

## Method validation

The fitness-for-purpose of the developed method was assessed by a single-laboratory method validation in accordance with Regulation (EC) No. 2002/657/EC [34]. The following performance characteristics of the developed method were investigated: selectivity, linearity, matrix effects, working range, limit of quantification (LOQ), repeatability, intermediate precision and trueness.

### Trueness assessment

The inaccuracies of the quantification of the peptide targets are partially due to the use of a universal sample preparation. Whilst a multi-analyte method seems to be an adequate approach for the quantification of the relevant peptide markers allowing the determination of the total milk protein content, the quantification of specific proteins usually requires dedicated sample preparations [21]. The experimental compromise selected for the extraction and digestion of the marker proteins may be the main contributor to the observed bias. Moreover, the baking process could induce changes in protein structures and interactions with other food ingredients, hence hindering the quantification of food proteins [35]. The peptide quantification can also be influenced by a biased calibrant preparation or peptide adsorption on vials.

Due to the lack of a reference material certified for its total milk protein content, whereby the individual mass fractions of the measured targets would be known, the TCMP results obtained applying the LC-MS method were compared with the TCMP results derived from the Dumas method. The TCMP mass added gravimetrically to the dough before baking the material was determined by the Dumas method on the SMP ingredient without an estimate of the corresponding measurement uncertainty. The ratio of the two results ranged from 75 to 90%. Figure 5 shows that the expanded ranges (*w*_TCMP_ ± U) overlap in approaches 2, 3 and 4. The same was observed for the data on the investigated cookies with the three incurred amount levels. Ultimately, this is an indication of the equivalence of the results of the methods but has limited use as a trueness estimator of the LC-MS method due to the inherent use of an arbitrary correction factor in the Dumas method.

The extraction efficiency was further assessed comparing the results obtained for an incurred material at 10.7 mg TCMP/kg baked cookie with the one obtained by spiking the SMP ingredient to the extracted blank cookie at the same level. A larger scatter of results was observed for the incurred material. The amount of IPA in the two samples was significantly lower than the amount of ALP (the two peptide markers of LACB). After exclusion of the IPA results, the agreement among the 10 signature peptides improved to reach a relative standard deviation (RSD) of 15% for the spiked material, which confirms the completeness of the digestion (except for IPA and LACB), and a RSD of 33% for the incurred sample.

The challenging quantification of the LACB protein was already observed by Nitride et al. [21]. The time course digestion experiments showed that the complete release of IPA was reached only after a digestion of 20 h. The differences observed between the incurred and the post-extraction spiked blank matrix may be due to the extraction and the baking process. The LACB protein is known to be relatively resistant to acidic hydrolysis and protease activities, not to mention the oxidation of the methionine residue in the ALP sequence, which may explain its high allergenic potential [35]. Johnson et al. [14] did not consider ALP as a reliable quantitative marker.

Matrix effects were investigated by spiking the mixture of peptide standards at increasing mass fractions (from 0.25 to 20 mg total milk protein per kg of test material) into the digested blank extract. The same methodology was used for preparing the calibrants with defined ratios. The plots of the resulting measurement data were linear for the eleven peptides (*R*^2^ = 0.99) over the entire tested range.

### Selectivity

Measurements using a blank matrix spiked either with natural or SIL peptides were evaluated to assess the degree of interferences in the measurement signals and hence the selectivity. No significant interferences were identified in the chromatograms for most of the acquired transitions. Some negligible interferences on labelled SIL peptides were observed for FFV, YLG and NAV, accounting for a maximum of 0.5% of the signals from natural analogue peptides. Furthermore, when comparing the blank matrix to the blank matrix spiked with peptides, no interferences were identified.

### Precision assessment

Repeatability was assessed by a three-factor nested design using an incurred material at 21.3 mg TCMP/kg of baked cookie. Three extracts from the material were digested each three times and analysed under repeatability conditions (same analyst, same instrument and same day). The results were evaluated using a one-way ANOVA at a 95% confidence level. The overall repeatability precision of the measurements and the individual contributions of extraction, digestion and instrumental analysis (MS measurement) steps are summarized in Table 4. Relative standard deviations for repeatability (RSD_r_) better than 7.5% were obtained for all peptides, except for AVP and YIP (RSD_r_ = 14.4% and 16.4%, respectively). Generally, the two main contributors to the repeatability assessment are the ‘extraction’ and the ‘instrumental analysis’. The contribution due to ‘digestion’ was only significant in the case of the LACB protein (see ALP, IPA).

**Table 4:** Relative standard deviation of repeatability and relative variance contributions of ‘extraction’, ‘digestion’ and ‘instrumental analysis’

Protein	Peptide	Extraction	Digestion	Instrumental	Total
CASA1	FFV	4.7%	0.0%	3.7%	6.0%
	YLG	2.2%	0.0%	3.5%	4.1%
CASA2	ALN	5.3%	0.7%	4.0%	6.7%
	FAL	6.4%	0.0%	3.5%	7.3%
	NAV	4.6%	0.0%	5.8%	7.4%
	VIP	5.6%	0.0%	4.8%	7.4%
CASB	AVP	7.3%	0.0%	12.4%	14.4%
	VLP	4.1%	2.8%	3.7%	6.2%
CASK	YIP	6.3%	0.0%	15.1%	16.4%
LACB	ALP	3.7%	3.9%	4.6%	7.1%
	IPA	2.2%	2.8%	4.0%	5.4%

Similarly, intermediate precision was assessed by a three-factor nested design, where the effect of analysts, days of analysis and extractions were investigated. Digestion was found to be a well-controlled parameter. Two extracts from an incurred cookie with nominal 10.7 mg TCMP/kg of baked cookie were digested by two analysts on two different days using the same instrument. The results were evaluated using a one-way ANOVA at a 95% confidence level. The intermediate precision of the measurements and the individual contributions are summarized in Table 5. Relative standard deviations for intermediate precision (RSD_ip_) are around 13% for most of all peptides. Exceptions were observed for FFV, YLG, ALN, AVP, YIP (all below 10%) and ALP (above 16%). Generally, the two main contributors to the intermediate precision are ‘extraction’ and ‘day of analysis’. The ‘analyst’ contribution is only significant in the case of ALP, NAV and VLP.

**Table 5:** Relative standard deviation of intermediate precision and relative variance contributions of ‘extraction’, ‘day of analysis’ and ‘analyst’

Protein	Peptide	Extraction	Day	Analyst	Total
CASA1	FFV	1.5%	3.2%	0.0%	3.5%
	YLG	7.1%	2.9%	0.0%	7.9%
CASA2	ALN	0.0%	8.9%	0.0%	8.9%
	FAL	4.2%	12.4%	0.0%	13.1%
	NAV	4.1%	9.7%	6.3%	12.3%
	VIP	6.6%	11.5%	0.0%	13.3%
CASB	AVP	0.0%	6.8%	0.0%	6.8%
	VLP	0.0%	6.8%	7.7%	10.3%
CASK	YIP	7.1%	0.0%	0.0%	7.1%
LACB	ALP	0.0%	15.5%	5.1%	16.3%
	IPA	3.4%	12.9%	0.0%	13.3%

### Linearity and LOQ

A calibration curve, ranging from 2.5 to 25 mg/kg (total milk protein), was constructed with seven equidistant calibration standards prepared from a natural blend working solution mimicking the natural protein abundances in milk. The labelled peptide (SIL) working solution blend was prepared at the middle mass fraction of the calibration curve. The matrix-matched calibration was conducted by spiking the seven calibration standards and a SIL peptide blend into the blank matrix before digestion to account for any modification or loss during the digestion and the clean-up step. The quantification was based on the measured signal ratios for the native (samples) and natural standard (calibration samples) to the SIL blend. A satisfactory linearity was observed for all eleven calibration curves (*R*^2^ ≥ 0.99). Five instrumental replicates were acquired for each calibration point. Despite the slight increase in the standard deviation of residuals observed with increasing molality, a linear regression model was used to estimate the molality of peptides in the baked cookies analysed.

The variations in the sensitivity of the method, i.e. the slope of the calibration function, for each peptide can be explained by different efficiencies of the ionization of the peptides derived from the same protein within the matrix background of the samples. As for the peptides derived from different proteins, the different sensitivities observed are due to the natural abundance of each protein in nature.

The limits of quantification of the complete procedure (LOQ) were calculated based on the standard deviation of six replicates of blank signals, obtained from incurred cookie (at nominal level of 2.7 mg SMP/kg baked cookie) samples having undergone the complete sample preparation protocol (including extraction and digestion). The LOQ was estimated as 10 times the standard deviation of the signal divided by the slope of the corresponding calibration curve [36]. The following LOQs were obtained (ordered by increasing value and expressed in mg TCMP/kg baked cookie): 0.15 for NAV; 0.16 (FFV); 0.42 (YLG); 0.53 (FAL); 0.70 (IPA); 1.5 (YIP); 1.8 (VIP); 1.9 (AVP); 1.9 (ALN); 3.9 (ALP) and 5.8 (VLP).

For consumer protection purposes, it may be more useful to evaluate the reported LOQs in the context of the VITAL 3.0 reference dose for milk (0.2 mg protein) and their respective reference amounts (portion size which could be consumed safely by 99% of allergic individuals). Peptides measurable with LOQs below 2 mg/kg, i.e. most of the markers above, could be used to assess products where an allergic individual is expected to consume a 100-g portion size or less. The peptides NAV and FFV could even be used for the assessment of products with a larger consumption size of up to 500 g.

## Conclusions

A reference method based on mass spectrometry has been developed following an analytical strategy relevant to the allergen measurement community. With the development of this reference method, the comparability of measurement results can be established. This method (i) allows a quantification based on a selection of representative allergenic peptide markers from the total milk protein; (ii) provides results in the common reporting unit ‘mg of total milk protein per kg of food’, in accordance with the latest agreements of the allergen community [5]; (iii) establishes the metrological traceability of the measurement results to the SI and (iv) is able to quantify the milk protein content at relevant clinical levels.

The method quantifies 11 peptide markers and determines the representative allergenic protein markers from the casein and whey fractions of the total milk protein. The peptide amounts are converted into the amount of protein and to the amount of total milk protein, logically expressed in ‘mg of total milk protein per kg of food’. The results are metrologically traceable to the SI via the use of well-characterized synthetic peptide standards for each peptide marker.

The method was developed and validated for incurred cookies including total milk protein at clinically relevant levels as requested by the allergen community. The fitness-for-purpose of this method is demonstrated by the suitable method performance characteristics obtained, such as LOQs mostly below 2 mg/kg. These parameters meet the quality requirements set by AOAC [37] for the quantification of the milk allergen content by mass spectrometry for the 11 marker peptides investigated.

The proof of concept presented, exemplified for the milk allergen, is the first step towards establishing reference methods for food allergen quantification. This method will be applied for the characterization of dedicated reference materials further facilitating the comparability of food allergen measurement results obtained by ELISA, mass spectrometry or other techniques targeting peptides or proteins.

## Electronic supplementary material

ESM 1 (PDF 95 kb).
