# Supplementary material for: A reference method for determining the total allergenic protein content in a processed food: the case of milk in cookies as proof of concept
Source: Anal Bioanal Chem. 2020 Oct 2;412(30):8249–67. doi: 10.1007/s00216-020-02959-0 (PMC7680749; doi:10.1007/s00216-020-02959-0)
Supplement: Supplementary file 1 — (PDF 95 kb). [file 216_2020_2959_MOESM1_ESM.pdf]

Analytical and Bioanalytical Chemistry

Electronic Supplementary Material

A reference method for determining the total allergenic protein content in a processed food: the case of milk in cookies as proof of concept

Maria José Martinez Esteso, Gavin O'Connor, Jørgen Nørgaard, Andreas Breidbach, Marcel Broheé, Elena Cubero-Leon, Chiara Nitride, Piotr Robouch, Hendrik Emons

Table S1 Peptide content in the standard solutions as determined by amino acid analysis (AAA), to be compared to the nominal content of 1 mg/g calculated from the peptides' purity as declared by the supplier

Protein	Peptide	Peptide. content (mg/g)	Standard uncertainty (u) mg/g	Relative uncertainty (u_{rel})
CASA1	FFV	0.67	0.01	1.1 %
CASA2	ALN	0.79	0.02	2.5 %
	FAL	0.60	0.01	1.4 %
	NAV	0.74	0.01	1.4 %
	VIP	0.44	0.01	1.2 %
CASB	AVP	0.72	0.01	1.2 %
	VLP	0.58	0.01	1.8 %
CASK	YIP	0.74	0.02	2.0 %
LACB	ALP	0.58	0.01	1.8 %
	IPA	0.70	0.01	1.1 %
